# DS-Mamba: Depthwise separable mamba for hyperspectral image classification

**DOI:** 10.1371/journal.pone.0342343

**Published:** 2026-03-12

**Authors:** Lin Wei, Huihan Yang, Yuping Yin, Zhiyuan Qu, Haonan Zheng

**Affiliations:** 1 Basic Teaching Department, Liaoning Technical University, Huludao, Liaoning, China; 2 School of Electronic and Information Engineering, Liaoning Technical University, Huludao, Liaoning, China; 3 Faculty of Electrical and Control Engineering, Liaoning Technical University, Huludao, Liaoning, China; Universidade Federal de Uberlandia, BRAZIL

## Abstract

Transformers experience quadratic computational complexity in hyperspectral image (HSI) classification tasks, which can result in error propagation and memory usage issues. Recently, Mamba architectures built upon the State Space Models have supplanted Transformers across various domains to accomplish long-range sequence modeling capability while demonstrating the advantages of linear computational efficiency. However, employing the basic Mamba model for HSI classification has problems associated with the extraction of spatial and spectral features. Motivated by this, we propose the DS-Mamba, a novel depthwise separable Mamba for HSI classification. Specifically, to extract the spatial and spectral features more efficiently, we design a depth spatial Mamba block (DSpaM), a depth spectral Mamba block (DSpeM) and a feature enhancement module. These blocks use depthwise separable convolution in conjunction with the basic Mamba block to improve classification accuracy while maintaining a low computational cost. Subsequently, to enhance the classification performance, feature weights are adjusted and spatial as well as spectral information are integrated through the feature fusion module. Finally, the feature information is enhanced and categorized by a classification module with Efficient Channel Attention (ECA). Through comparative experiments, DS-Mamba achieved overall accuracies of 96.54%, 91.52%, and 94.89% on the Pavia University, Hanchuan, and Houston datasets, respectively. Its classification performance surpassed that of several advanced transformer-based methods. Furthermore, DS-Mamba has lower model parameters and floating point operations (FLOPs), with only 137.74K parameters and 12.52G FLOPs recorded on the Pavia University dataset.

## 1 Introduction

Hyperspectral images (HSI) capture information regarding the electromagnetic radiation reflected by objects across continuous narrow bands [1,2], which enables precise identification and classification of objects. Due to its advantages, HSI has found extensive applications aross various remote sensing scenarios, such as geological resource exploration [3], environmental monitoring [4] and precision agriculture [5]. As a fundamental task of HSI processing, the core objective of HSI classification is to distinguish features at the pixel level [6].

Early research methodologies commonly employed techniques such as support vector machine (SVM) [7], principal component analysis (PCA) [8], and linear discriminant analysis (LDA) [9] for feature extraction or dimensionality reduction. These methods primarily concentrate on leveraging the spectral features of HSI while overlooking the spatial information. Therefore, some researchers have developed classification frameworks based on spectral spatial features, such as extended morphological profiles (EMP) [10], extended multi-attribute profiles (EMAP) [11], and sparse manifold representations [12]. However, these methods rely on manually designed features and predetermined parameters, which are insufficient for effectively capturing feature information in complex environments.

In recent years, deep learning has been widely used in the field of computer vision [13,14], and it has also propelled advancements in HSI classification research [15]. The primary classical deep learning models encompass convolutional neural networks (CNN) [16], recurrent neural networks (RNN) [17], graph convolutional networks (GCN) [18], and Transformers [19]. Among these, CNN architectures have garnered significant attention in research due to their local receptive fields and the properties of parameter sharing. The 2D-CNN proposed by Lee et al. [20] uses multiple convolutional and pooling layers to extract deep features, but the structure of the deep full convolution results in a relatively high number of parameters and computational complexity. Zhong et al. [21] developed an end-to-end residual network utilizing 3D-CNN, which effectively captures deep spectral-spatial information directly from the original 3D HSI cube. However, the operation of 3D convolution significantly escalates the computational demands of the model. The HybridSN proposed by Roy et al. [22] integrates 2D and 3D convolution, reducing the complexity of the model. Li et al. [23] constructed a depth-separable residual neural network (ResNet), which separates the spectral and spatial information using depthwise separable convolution and reduces the network size to mitigate the risk of overfitting. However, CNN-based models have limited ability to model global context, and fixed convolutional kernels pose challenges in adapting to dynamically changing input features. When training data is scarce, these models are susceptible to overfitting, resulting in diminished generalization capabilities. Recently, the Transformer has been widely applied owing to its powerful capability in modeling long-range dependencies. He et al. [24] proposed a cross-spectral vision transformer (CSiT), which employs a dual-branch architecture to extract pixel-level multi-scale features. Moreover, Sun et al. [25] introduced a spectral-spatial feature tokenization transformer (SSFTT) that leverages the strengths of both CNN and Transformer. This model employs 2D and 3D convolutions to extract shallow features, subsequently incorporating a Gaussian-weighted feature tokenizer for feature transformation, which generates the input tokens required for the Transformer block. Hon et al. [26] introduced a SpectralFormer to produce grouped spectral embeddings by learning spectrally localized sequence information from neighboring bands. MorphFormer proposed by Roy et al. [27] employs spectral and spatial morphological convolution to improve the interaction between structure and shape information. The cross spatial-spectral dense transformer (CS2DT) [28] utilizes an adaptive dense encoder to extract multi-scale semantic information and employs cross-attention mechanisms for effective feature fusion. In addition, [29] designed a lightweight network, GSC-ViT, which uses grouped separable convolution to decrease the number of parameters while effectively capturing local spectral-spatial information.

However, the secondary computational complexity brought by the self-attention mechanism of Transformers may lead to inefficiency and memory limitations when dealing with high-dimensional data from HSI. Recently, the Mamba [30] built on the state-space model (SSM) has shown excellent performance in natural language processing (NLP) tasks. By introducing a selective scanning mechanism and hardware-aware algorithm, Mamba exhibits the advantages of linear computational efficiency while enabling remote modeling, which is anticipated to serve as an alternative to Transformer. Consequently, several studies have commenced the application of Mamba models for computer vision tasks. Vim [31] employs positional embedding to annotate image sequences and models state space representations using bidirectional compressed vision. VMamba [32] collects contextual information through four distinct scanning routes, drawing from a diverse array of sources and perspectives. They demonstrate outstanding performance in tasks such as image classification and segmentation. However, Mamba is less frequently employed in HSI classification tasks. To this end, we proposes a deepthwise separable Mamba (DS-Mamba) for HSI classification. The main contributions are summarized as follows:

1) By integrating depthwise separable convolution with Mamba, a depth spatial Mamba block and a depth spectral Mamba block are developed to effectively extract both spatial and spectral features. This approach significantly reduces the computational complexity of the model, thereby alleviating the overall computational burden.
2) A feature fusion module has been developed to integrate the extracted spatial and spectral information by adjusting the weights accordingly. Additionally, the concept of residual learning is incorporated through the use of skip connections, which enhances both model performance and generalization capability.
3) The lightweight Efficient Channel Attention (ECA) [33] is introduced prior to classification, facilitating local cross-channel interactions, thereby enhancing the representation of features.

## 2 State space models and Mamba

The State Space Model (SSM) [34] originates from continuous linear time-invariant systems and is widely used to model dynamic systems through the state variables. SSM converts an input one-dimensional signal x(t)∈R into an output y(t)∈R through an intermediate hidden state $h(t)\in R^{N}$. This process can be expressed through a linear ordinary differential equation as:


$$\begin{array}{c} h^{\prime }(t)=Ah(t)+Bx(t)\; \end{array}$$ (1)

$$\begin{array}{c} y(t)=Ch(t) \end{array}\mathrm{\backslash ]}$$ (2)

where $h^{\prime }(t)\in R^{N}$ denotes the time derivative of h(t), $A\in R^{N\times N}$ represent the state matrix, and $B\in R^{N\times 1}$ and $C\in R^{N\times 1}$ signify the projection matrices.

However, SSM, as a continuous time system, is difficult to be directly integrated into deep learning algorithms. Consequently, discretization is achieved through the application of the zero-order hold (ZOH) technique along with specified time scales. This process transforms the continuous parameters A and B into discrete parameters $\overset{―}{A}$ and $\overset{―}{B}$:


$$\begin{array}{c} \overset{―}{A}=e^{\Delta A}\; \end{array}\mathrm{\backslash ]}$$ (3)

$$\begin{array}{c} \overset{―}{B}={(\Delta A{)}^{-1}(e}^{\Delta A}-I)•\Delta B\; \end{array}\mathrm{\backslash ]}$$ (4)

where Δ is the time scale parameter. The discretized SSM can be expressed through a linear ordinary differential equation as


$$\begin{array}{c} h_{t}=\overset{―}{A}h_{t-1}+\overset{―}{B}x_{t}\; \end{array}\mathrm{\backslash ]}$$ (5)

$$\begin{array}{c} y_{t}=Ch_{t}\; \end{array}$$ (6)

The above computation can be expressed in terms of the global convolution operation as:


$$\begin{array}{c} \overset{―}{K}=(C\overset{―}{B},C\overset{―}{AB},\ldots ,C{\overset{―}{A}}^{L-1}\overset{―}{B}) \end{array}$$ (7)

$$\begin{array}{c} y=x*\overset{―}{K} \end{array}$$ (8)

where L denotes the length of the input sequence x, $\overset{―}{K}\in R^{L}$ represents the dimensions of the structured convolution kernel, and * is convolution operation.

**Fig 1:**
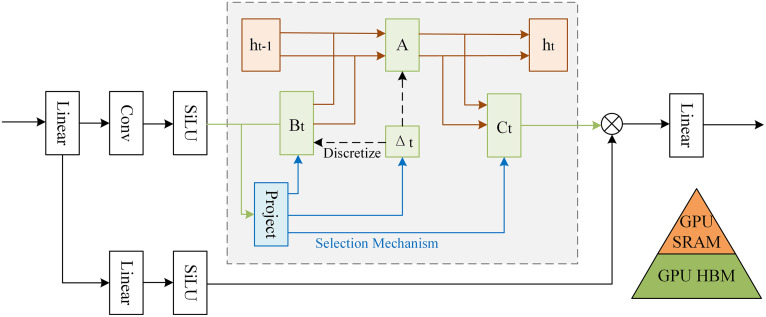
The structure of Mamba.

The traditional SSM struggles to effectively capture the contextual information within input sequence [26]. To address this limitation, Mamba introduces a distinctive selection mechanism that enables the model to dynamically adjust the parameters of the SSM according to the input data. This mechanism allows for selective retention or discarding of context-awareness in relation to the sequence state, thereby enhancing its capability to process long sequential data. Additionally, Mamba introduces a hardware-aware algorithm designed to enhance the computational efficiency of the model. The parameters of SSM (Δ, A, B, C) are loaded in fast Static Random-Access Memory (SRAM) instead of slower High Bandwidth Memory (HBM). A series of pre-processing steps, such as discretization, are conducted in SRAM before the final output is written back to HBM, which improves the training efficiency of the model. The detailed architecture of Mamba is shown in Fig 1.

## 3 Model topology

**Fig 2:**
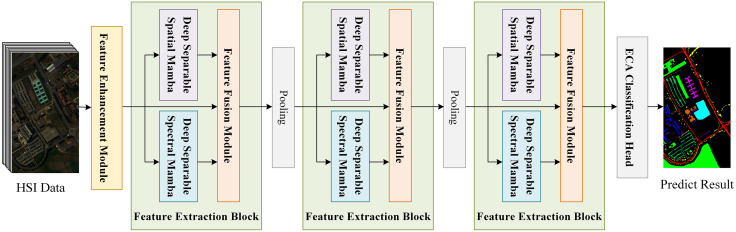
The network structure of DS-Mamba.

In this section, the design of DS-Mamba model will be presented. The overall framework is illustrated in Fig 2. The model has three main components: feature enhancement module, feature extraction module, and ECA classification module. Different from the traditional patch input-based model, the image input of this model first passes through the feature enhancement module to extract information of each pixel. This fine-grained pixel embedding enables the model to capture more intricate local features, thereby demonstrating improved accuracy and robustness in HSI tasks. Then the spatial and spectral features are extracted and feature fusion is performed by the feature extraction module, respectively. Specifically, the feature extraction module mainly contains three parts: depth spatial Mamba block (DspaM), depth spectral Mamba block (DSpeM) and feature fusion module. Finally, the ultimate image classification is conducted following the classification head containing ECA attention.

### 3.1 Feature enhancement module

**Fig 3:**
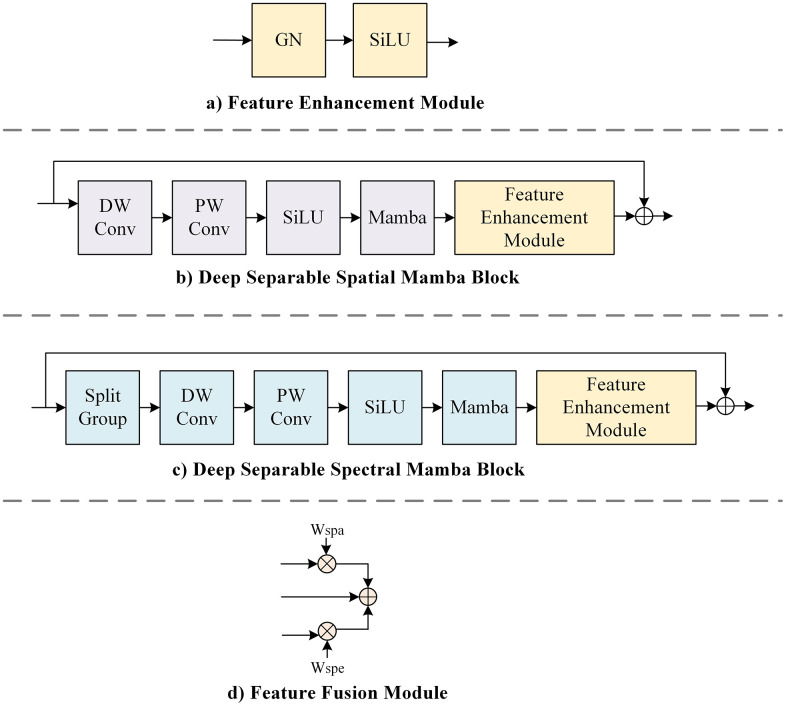
Details of the module.

The feature enhancement module, as illustrated in Fig 3(a), aims to construct more discriminative features in the spectral domain. Specifically, the group normalization mitigates the dependence on batch size by normalizing the feature maps within each group, thereby demonstrating enhanced stability during training with small batch samples. The SiLU activation function improves the nonlinear representation of the model through its smoothness and self-gating mechanism, while also accelerating model convergence. FEM not only allows the model to capture subtle variations in spectral information but also significantly enhances the discrimination and robustness of features.

### 3.2 Depth spatial Mamba block

Although existing Transformer-based models demonstrate exceptional capabilities in long-range modeling, their quadratic computational complexity results in significant error propagation within the model. Furthermore, the extensive number of parameters and high computational demands also lead to issues related to memory usage. To address this issue, we design DspaM using depthwise separable convolution to minimize the computational effort and the number of parameters. Simultaneously, DspaM utilizes the Mamba as the basic feature extraction unit to build long-range dependencies in linear computational efficiency. The detailed structure of the DSpaM is shown in Fig 3(a). The forward process can be formulated as follows:


$$\begin{array}{c} X_{2}=\text{ Mamba}\left(\text{SiLU}\left(\text{PConv}\left(\text{DConv}\left({\text{X}}_{\text{1}}\right)\right)\right)\right)\; \end{array}$$ (9)

$$\begin{array}{c} X_{3}=FEM\left(X_{2}\right)=SiLU\left(GN\left(X_{2}\right)\right) \end{array}$$ (10)

where DConv, PConv, GN, SiLU, and FEM denote the depthwise convolution with 3 × 3 kernel size, the pointwise convolution with 1 × 1 kernel size, the group normalization (GN) layer, SiLU activation function, and feature enhancement module, respectively. The Mamba denotes the standard Mamba block proposed in [30].

### 3.3 Depth spectral Mamba block

Each pixel in a HSI encompasses hundreds of consecutive spectral bands, which exhibit complex interactions and dependencies. How to effectively model the relationships among these spectral bands and extract discriminative features is a critical challenge. Therefore we propose a DSpeM to effectively harnesses the abundant spectral information of HSI. The details are illustrated in Fig 3(b). The input features are first grouped for spectral dimensions, then fed into the Mamba block after depth separable convolution and SiLU activation function, and finally go through the feature enhancement module. The DSpeM is computed as follows:


$$\begin{array}{c} X_{5}=SplitGroup\left(X_{4}\right) \end{array}$$ (11)

$$\begin{array}{c} X_{6}=\text{ Mamba}\left(\text{SiLU}\left(\text{PConv}\left(\text{DConv}\left({\text{X}}_{\text{5}}\right)\right)\right)\right) \end{array}$$ (12)

$$\begin{array}{c} X_{7}=FEM\left(X_{6}\right)=SiLU\left(GN\left(X_{6}\right)\right) \end{array}$$ (13)

### 3.4 Feature fusion module

In HSI classification, the effective integration of spatial and spectral information can provide more accurate identification and classification of ground objects, thus improving the overall performance of the model. This motivates us to design a feature fusion module. As depicted in Fig 3(d), we employ jump connections and weighting to fuse spatial and spectral information while mitigating gradient vanishing and overfitting. The weights W are learned and updated through backpropagation after random initialization.

### 3.5 ECA classification module

**Fig 4:**
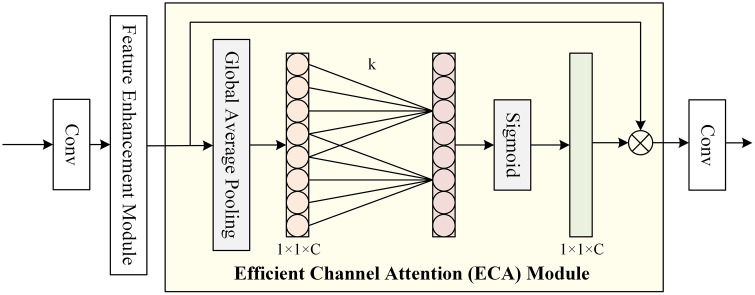
ECA classification module.

We design the ECA mechanism within the classification head, as shown in Fig 4, with the aim of capturing inter-channel dependencies prior to classification and enhancing feature representation. ECA implements a local cross-channel interaction strategy without dimensionality reduction through 1D convolution to enhance performance while maintaining lower model complexity. ECA adaptively determines the size of the convolution kernel k by the channel dimensions C in order to determine the local cross-channel interaction coverage. Initially, input features are processed through a dot convolution and feature enhancement module, followed by global average pooling to obtain aggregated features. Subsequently, these features undergo 1D convolution to generate channel weights, which are then passed through a sigmoid activation function before being subjected to 2D convolution for classification purposes.

## 4 Experimental results and analysis

### 4.1 Datasets description

In order to comprehensively evaluate the performance of the proposed model, considering the diversity of spectral information, spatial resolution, and scene types, three widely used datasets are selected: the Pavia University, the WHU-Hi-HanChuan (HanChuan) [35] and the Houston.

1) The Pavia University dataset was collected from the University of Pavia campus in Italy, with an image size of 610 × 340 pixels and a spatial resolution of 1.3 m. The HSI image contains 103 bands with a wavelength range approximately from 0.43 µm to 0.86 µm. The dataset contains nine distinct land use categories. The Pavia University dataset is a classic dataset commonly used for HSI classification. It serves as an effective benchmark for evaluating the performance disparities between novel models and traditional approaches. The detailed category information is presented in Table 1.

**Table 1:** Category details of the Pavia University dataset.

Index	Category	Train	Validation	Test	Total
**0**	Asphalt	30	10	6591	6631
**1**	Meadows	30	10	18609	18649
**2**	Gravel	30	10	2059	2099
**3**	Trees	30	10	3024	3064
**4**	Metal sheets	30	10	1305	1345
**5**	Bare soil	30	10	4989	5029
**6**	Bitumen	30	10	1390	1330
**7**	Bricks	30	10	3642	3682
**8**	Shadows	30	10	907	947

2) The Hanchuan dataset was acquired in 2016 in Hanchuan area, Hubei Province, China, by an airborne remote sensing platform with an image size of 1217 × 303 pixels, with a spatial resolution of approximately 0.109 meters. There were 274 bands in the wavelength range of 0.4 µm to 1 µm. The study area is a combination of urban and rural areas, containing 16 diverse land cover types such as buildings, water bodies, cultivated land, and crops, mainly agricultural scenes, filling the gap in agricultural hyperspectral analysis. Especially, the spectral similarity of farmland land cover types is high, making it suitable for testing the feature learning and discrimination capabilities of models. An overview of this dataset is given in Table 2.

**Table 2:** Category details of the HanChuan dataset.

Index	Category	Train	Validation	Test	Total
**0**	Strawberry	30	10	44695	44735
**1**	Cowpea	30	10	22713	22753
**2**	Soybean	30	10	10247	10287
**3**	Sorghum	30	10	5313	5353
**4**	Water spinach	30	10	1160	1200
**5**	Watermelon	30	10	4493	4533
**6**	Greens	30	10	5863	5903
**7**	Trees	30	10	17938	17978
**8**	Grass	30	10	9429	9469
**9**	Red roof	30	10	10476	10516
**10**	Gray roof	30	10	16871	16911
**11**	Plastic	30	10	3639	3679
**12**	Bare soil	30	10	9076	9116
**13**	Road	30	10	18520	18560
**14**	Bright object	30	10	1096	1136
**15**	Water	30	10	75361	75401

3) The Houston dataset, which covers the University of Houston and surrounding regions in Texas, USA, is the official dataset of the 2013 IEEE Geoscience and Remote Sensing Society (GRSS) Data Fusion Contest and is authoritative for evaluating model performance. It features an image size of 349 × 1905 pixels and a spatial resolution of 2.5 meters. The hyperspectral modal contains 144 spectral bands covering the spectral range from 0.38 to 1.05 µm. The scene encompasses 15 typical urban features with high spectral mixing, rendering it an ideal setting for evaluating the model’s generalization ability in complex environments. The detailed category information is shown in Table 3.

**Table 3:** Category details of the Houston dataset.

Index	Category	Train	Validation	Test	Total
**0**	Healthy Grass	30	10	1211	1251
**1**	Stressed Grass	30	10	1214	1254
**2**	Synthetic Grass	30	10	657	697
**3**	Tree	30	10	1204	1244
**4**	soil	30	10	1202	1242
**5**	Water	30	10	285	325
**6**	Residential	30	10	1228	1268
**7**	Commercial	30	10	1204	1244
**8**	Road	30	10	1212	1252
**9**	Highway	30	10	1187	1227
**10**	Railway	30	10	1195	1235
**11**	Parking Lot 1	30	10	1193	1233
**12**	Parking Lot 2	30	10	429	469
**13**	Tennis Court	30	10	388	428
**14**	Running Track	30	10	620	660

### 4.2 Experimental setup and evaluation metrics

The training and testing environment of this study was established on the PyTorch 2.1.2 framework, which was accelerated with CUDA 11.8. The Adam optimizer was used to train the model with a learning rate of 0.0003. 30 and 10 samples were randomly selected for the training and validation sets, respectively, and the test set was made up of the rest. The following five generalized performance evaluation metrics were used in this experiment: Overall Accuracy (OA), Average Accuracy (AA), Kappa coefficient, Parameters, and floating point operations (FLOPs). To ensure the fairness of the comparison, all experiments are carried out under the identical experimental conditions, and the results are taken as the mean and standard deviation of ten consecutive experiments. Hardware environment: the operating system is Windows Subsystem for Linux subsystem of Windows 11, the processor is Intel Core i7-14700HX CPU, the graphics card is NVIDIA GeForce RTX 4070 GPU with 8GB of VRAM, and 16GB of system RAM.

### 4.3 Comparison experiments and analysis

To demonstrate the effectiveness of DS-Mamba, we select three representative classification methods for comparison, including the traditional method, the CNN-based method and the Transformer-based method. The details can be listed as the following:

1) SVM [7]: The model adopts SVM (support vector machine) for HSI classification.
2) 2D-CNN [16]: The model mainly consists of 2D convolution, maximum pooling and fully connected layers.
3) HybridSN [21]: The model uses a mixture of 2D and 3D convolution to extract spatial and spectral information, which mainly consists of 2D convolution, 3D convolution and fully connected layers.
4) SpectralFormer [23]: The model generates grouped spectral embeddings by learning the spectral sequence information of adjacent bands, which mainly consists of grouped spectral embedding layer, cross-layer adaptive fusion, transformer layer, and an MLP (Multilayer Perceptron) for classification.
5) MorphFormer [24]: The model fuses attention and morphological features, which mainly consists of spectral spatial morphological convolution, attention module based on Morphological Feature Fusion (MFF), and linear layer for classification.
6) GSC-ViT [25]: The model adopts a groupwise separable convolution ViT to capture local and global spatial spectral information, which mainly consists of group-separable convolutional blocks, group-separable multi-head self-attention, global average pooling layer and softmax classifier.

**Fig 5:**
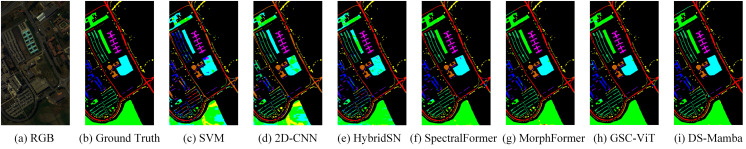
Qualitative visualization of the classification map for the Pavia University dataset.

**Fig 6:**
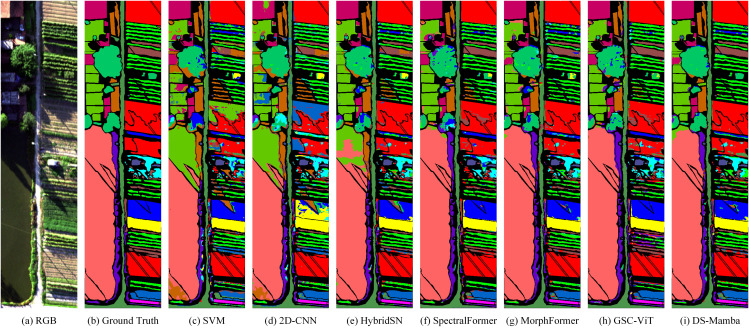
Qualitative visualization of the classification map for the HanChuan dataset.

**Fig 7:**
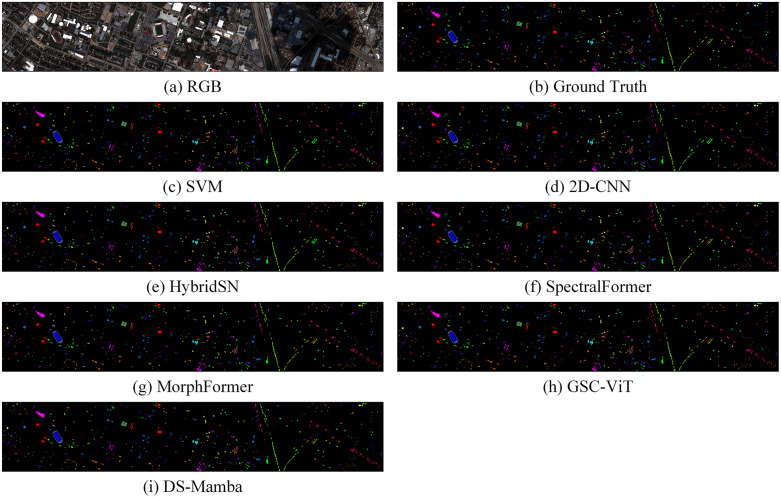
Qualitative visualization of the classification map for the Houston dataset.

**Fig 8:**
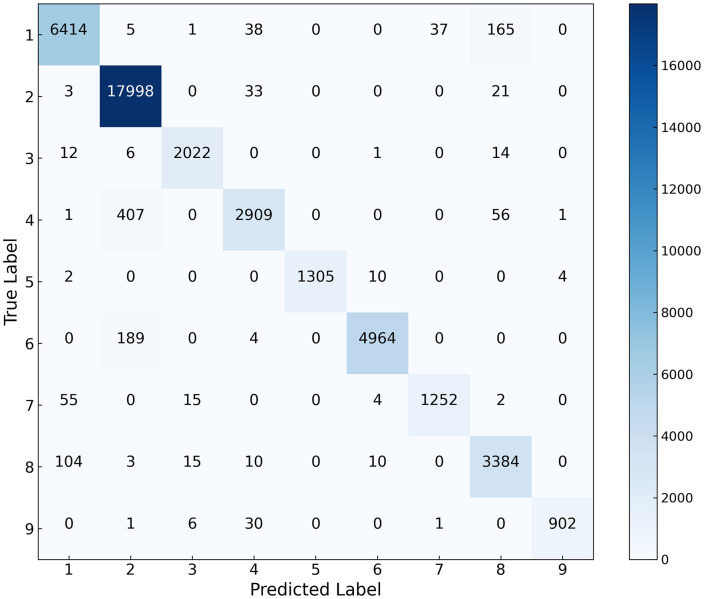
Confusion matrix of the Pavia University dataset.

**Fig 9:**
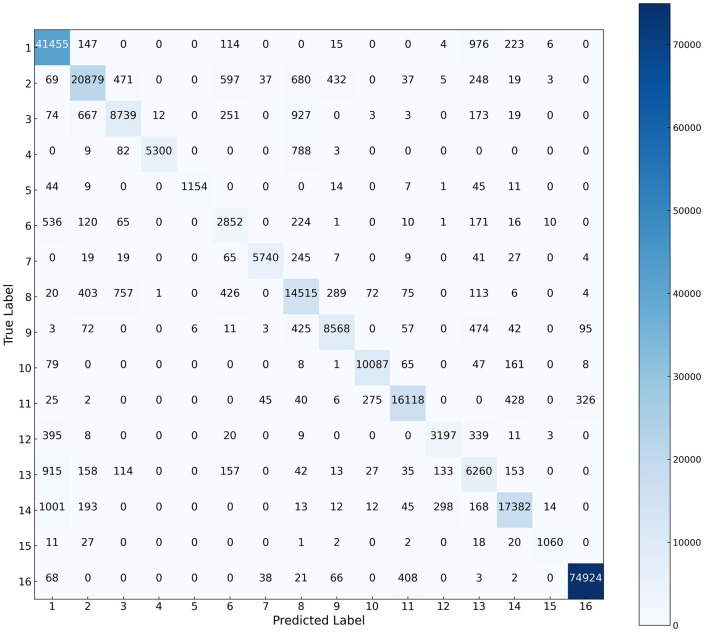
Confusion matrix of the HanChuan dataset.

**Fig 10:**
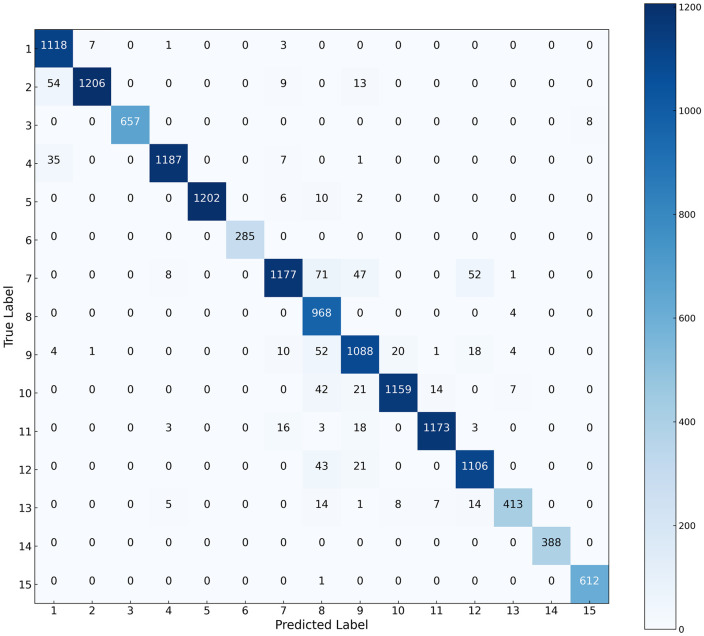
Confusion matrix of the Houston dataset.

The classification results and complexity of various models across the three datasets are presented in Tables 4–6 and Figs 5–7, while the confusion matrices on the test sets are illustrated in Figs 8–10. It can be observed that the proposed DS-Mamba demonstrates superior performance compared to other methods. From what is seen in Tables 4–6, the classification accuracy of DS-Mamba is improved more significantly and is the highest compared to other networks.

**Table 4:** Quantitative Comparison results of the Pavia University dataset.

Metrics	SVM	2D-CNN	HybridSN	SpectralFormer	MorphFormer	GSC-ViT	DS-Mamba
**OA (%)**	72.27 ± 2.98	75.34 ± 1.86	80.21 ± 2.47	90.16 ± 2.57	91.34 ± 2.11	92.65 ± 2.30	**96.54 ± 1.15**
**AA (%)**	72.96 ± 1.37	73.25 ± 1.41	77.13 ± 1.97	79.93 ± 2.67	85.47 ± 1.92	91.89 ± 1.73	**96.43 ± 0.97**
**Kappa (%)**	64.83 ± 3.06	70.63 ± 3.62	74.59 ± 3.43	77.40 + 4.41	84.52 ± 3.83	90.38 ± 2.95	**97.25 ± 2.15**
**Paramters(K)**		1484.55	5121.27	167.27	148.97	77.9	137.74
**FLOPs (G)**		3199.74	5548.16	9369.15	2400.9	2222.85	12.52

**Table 5:** Quantitative comparison results of the HanChuan dataset.

Metrics	SVM	2D-CNN	HybridSN	SpectralFormer	MorphFormer	GSC-ViT	DS-Mamba
**OA (%)**	60.98 ± 1.35	68.92 ± 1.78	74.88 ± 3.02	82.99 ± 2.31	82.76 ± 2.54	83.14 ± 3.96	**91.25 ± 0.91**
**AA (%)**	51.48 ± 1.46	56.45 ± 1.15	70.47 ± 1.62	71.13 ± 2.56	72.47 ± 2.64	73.97 ± 2.06	**89.8 ± 0.7**
**Kappa (%)**	56.00 ± 1.49	63.41 ± 1.86	72.49 ± 1.36	80.29 ± 2.6	80.49 ± 3.55	80.57 ± 4.33	**89.73 ± 1.62**
**Paramters(K)**		1897.44	9619.58	557.1	258.8	122.13	160.53
**FLOPs (G)**		12047.87	29315.33	61601.08	9299.08	6150.93	30.3

**Table 6:** Quantitative comparison results of the Houston dataset.

Metrics	SVM	2D-CNN	HybridSN	SpectralFormer	MorphFormer	GSC-ViT	DS-Mamba
**OA (%)**	79.63 ± 1.04	82.95 ± 1.67	84.79 ± 2.26	89.13 ± 2.12	82.76 ± 2.54	91.85 ± 1.05	**94.89 ± 0.73**
**AA (%)**	80.70 ± 1.03	82.24 ± 1.52	83.49 ± 1.68	89.89 ± 1.74	72.47 ± 2.64	92.97 ± 0.92	**95.91 ± 0.68**
**Kappa (%)**	77.97 ± 1.12	80.25 ± 1.91	82.46 ± 2.51	88.24 ± 2.29	80.49 ± 3.55	91.18 ± 1.14	**94.64 ± 2.04**
**Paramters(K)**		1579.79	7223.3	229.04	157.74	88.78	143.76
**FLOPs (G)**		12823.59	20747.78	44871.62	9084.88	8126.23	43.61

On the Pavia University dataset, compared with GSC-ViT, the OA, AA and Kappa of DS-Mamba increased by 3.89%, 4.54% and 6.87% respectively. On the Hanchuan dataset, the classification results of the three transformer-based models were comparable, and DS-Mamba demonstrated superior accuracy, with an increase in OA of approximately 8%, AA by around 17%, and Kappa by about 9%. On the Houston dataset, compared with the second-place GSC-ViT, DS-Mamba improved OA, AA and Kappa by approximately 3%. After conducting ten repeated experiments, the fluctuations in OA, AA, and Kappa for DS-Mamba on the Hanchuan dataset were recorded at only 0.91%, 0.7%, and 1.62%, respectively. These values are notably 2% to 3% lower than those observed for other models, indicating that our model exhibits greater stability. From what is seen in Figs 5–7, the proposed DS-Mamba demonstrates a closer alignment with the ground truth map. All other networks suffer from more classification errors, high noise, and unclear boundaries.

**Fig 11:**
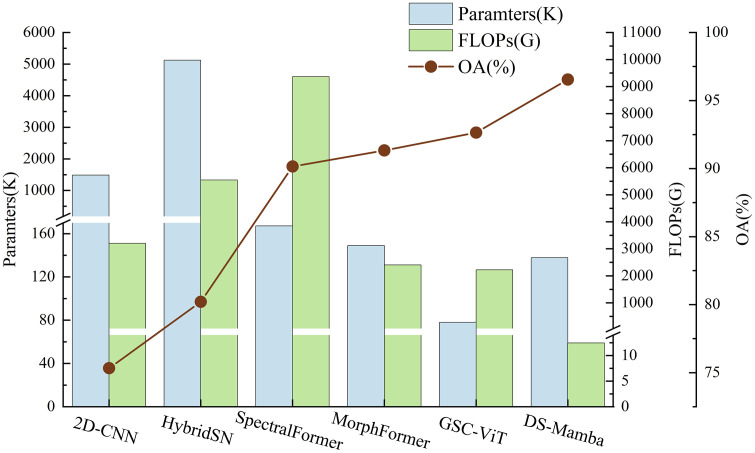
Comparison of complexity and OA of the Pavia University Dataset.

Meanwhile, DS-Mamba has fewer parameters and exhibits significantly lower computational intensity compared to the other models. Although the parameter count of DS-Mamba is slightly higher than that of GSC-ViT across all three datasets, its FLOPs are considerably reduced. On the Pavia University dataset, DS-Mamba demonstrated a modest increase in parameter count of only 59K compared to GSC-ViT, while achieving a significant reduction in FLOPs by 2210G. On the Hanchuan dataset, DS-Mamba not only decreased the parameter count by 98K but also decreased FLOPs by an impressive 9269G when compared to MorphFormer. When contrasted with CNN-based models, both the parameter count and computational costs were markedly diminished. On the Houston dataset, relative to HybridSN, DS-Mamba achieved a substantial reduction in parameters amounting to 4984M and a decrease in FLOPs by 5536G. This efficiency is attributed to the linear computational complexity inherent in Mamba. The comparison of the complexity and OA of each model is presented in Fig 11, where DS-Mamba demonstrates superior classification accuracy while maintaining lower model complexity. This further demonstrates the efficacy of the proposed method.

### 4.4 Ablation experiments and analysis

To evaluate the effectiveness of the key components of the models, the ablation experiments were conducted on all three dataset. The parameter settings and training strategies were consistently maintained across all models, with results reported as the mean and standard deviation derived from ten consecutive experiments. Four comparison models have been selected: DS-Mamba-0 (using only DspaM in the feature extraction module), DS-Mamba-1 (using only DSpeM in the feature extraction module), DS-Mamba-2 (using the normal classification header instead of the one that incorporates ECA), and DS-Mamba-3(removing the Feature Enhancemen Module). The experimental results are shown in Tables 7–9. Compared with DS-Mamba, the accuracy of both DS-Mamba-0 and DS-Mamba-1 have decreased to varying degrees. On the Hanchuan dataset, OA, AA, and Kappa have all declined by approximately 4%. This indicates that both spatial and spectral information hold significant importance. On the Pavia University dataset and the Houston dataset, we observed that the classification accuracy of DS-Mamba-0 was 1% to 2% higher than that of DS-Mamba-1, indicating that spatial features are more distinguishable than spectral features. Compared with DS-Mamba-2, DS-Mamba only increased the parameter and FLOPs by 6 and 1.4M respectively on the Pavia University dataset, but the OA, AA, and Kappa were improved by about 1.5%, 1%, and 3%, respectively. This indicates that the ECA classification module can bring significant performance gains while introducing very few additional parameters and negligible computations. Compared with DS-Mamba, the OA, AA, and Kappa of DS-Mamba-3 decreased by 3% to 8%, thereby demonstrating the efficacy of the feature enhancement module. This is due to the fact that Group Normalization can enhance the model’s generalization capability by minimizing dependence on batch computations. Additionally, the SiLU activation function helps mitigate the vanishing gradient problem. Consequently, the feature enhancement module contributes to improved training stability, augments the model’s expressive power, and ultimately leads to more accurate classification outcomes for the model.

**Table 7:** Results of ablation experience of the Pavia University dataset.

Metrics	DS-Mamba-0	DS-Mamba-1	DS-Mamba-2	DS-Mamba-3	DS-Mamba
**OA (%)**	94.05 ± 1.55	92.64 ± 1.77	94.92 ± 1.2	91.38 ± 2.87	96.54 ± 1.15
**AA (%)**	95.17 ± 1.15	93.15 ± 0.85	95.24 ± 1.14	93.11 ± 1.7	96.43 ± 0.97
**Kappa (%)**	95.25 ± 2.39	94.51 ± 2.07	94.63 ± 3.16	92.5 ± 3.79	97.25 ± 2.15
**Parameters (K)**	84.365	84.365	137.735	137.741	137.741
**FLOPs (G)**	7.7436	7.7436	12.5148	12.5164	12.5164

**Table 8:** Results of ablation experience of the Hanchuan dataset.

Metrics	DS-Mamba-0	DS-Mamba-1	DS-Mamba-2	DS-Mamba-3	DS-Mamba
**OA (%)**	86.83 ± 1.78	88.12 ± 1.19	89.27 ± 0.75	85.49 ± 1.49	91.25 ± 0.91
**AA (%)**	85.67 ± 1.18	85.99 ± 1.44	87.19 ± 1.36	81.39 ± 1.57	89.8 ± 0.7
**Kappa (%)**	84.2 ± 2.83	84.6 ± 3.09	88.06 ± 3.42	81.27 ± 3.37	89.73 ± 1.62
**Parameters (K)**	107.156	107.156	144.016	160.532	160.532
**FLOPs (G)**	21.8469	21.8496	29.9253	30.3018	30.3018

**Table 9:** Results of ablation experience of the Houston dataset.

Metrics	DS-Mamba-0	DS-Mamba-1	DS-Mamba-2	DS-Mamba-3	DS-Mamba
**OA (%)**	93.65 ± 1.3	92.48 ± 1.54	93.67 ± 1.26	88.33 ± 1.40	94.89 ± 0.73
**AA (%)**	94.5 ± 1.16	93.59 ± 1.26	94.81 ± 1.31	89.94 ± 1.33	95.91 ± 0.68
**Kappa (%)**	93.71 ± 2.02	92.85 ± 2.17	93.57 ± 1.92	89.00 ± 1.95	94.64 ± 2.04
**Parameters (K)**	90.387	90.387	127.247	143.763	143.763
**FLOPs (G)**	28.3341	28.3341	42.9212	43.6051	43.605

### 4.5 Discussion

Through experiments, we found that the Transformer-based model demonstrates superior classification performance compared to the CNN-based model. CNNs require stacking multiple convolutional kernels to expand their receptive fields, which limits their ability to effectively capture global context and necessitates a fixed input size. In contrast, the self-attention mechanism inherent in Transformers endows them with robust long-range modeling capabilities and allows for flexible adjustment of input sizes through positional encoding. However, the self-attention computation in Transformers relies on weight matrix multiplication, introducing quadratic computational complexity that limits its efficiency in hyperspectral image classification.

The proposed DS-Mamba is an advancement based on Mamba, characterized by linear computational complexity and fewer parameters and computational costs compared to Transformers. DS-Mamba not only efficiently captures long-range dependencies but also dynamically adjusts weights through a selection mechanism, enabling it to extract more detailed feature information. The model utilizes a dual-branch architecture to conduct in-depth modeling of spatial and spectral information independently, thereby fully accommodating the two-dimensional spatial characteristics and one-dimensional spectral sequence properties inherent in HSI. This design ensures a complementary relationship between spatial and spectral features. The Deep Spatial Mamba block effectively addresses local spatial discontinuities that arise from one-dimensional scanning, while the Deep Spectral Mamba block is dedicated to capturing fine-grained discriminative features within the spectral dimension. Consequently, this enables the model to simultaneously consider both local details and global dependencies. Deep separable convolutions are employed to model spatial features for each channel without significantly increasing the number of parameters; subsequently, cross-channel information is fused through pointwise convolutions. This approach enhances feature extraction efficiency while preserving rich discriminative information. The introduced feature fusion module dynamically adjusts feature weights during the integration of spatial and spectral data, thereby enhancing the model’s focus on critical information. Incorporating an ECA mechanism into the classification head emphasizes discriminative features across key channels through effective local cross-channel interactions. Compared to traditional attention mechanisms, ECA improves fine-grained classification accuracy without imposing significant computational overhead. Comparative experiments conducted on three representative datasets demonstrate that DS-Mamba consistently outperforms CNN- and Transformer-based models across all evaluated metrics: OA, AA, and Kappa coefficient. Notably, it achieves these results with substantially fewer parameters and reduced computational resources compared to Transformer-based models. Ablation studies further validate the effectiveness of the proposed modules.

However, this study does have certain limitations. It did not account for hierarchical feature extraction, which may hinder the full utilization of contextual information across different levels, particularly when category differences are subtle or feature scales vary significantly. This oversight can result in suboptimal classification performance in complex scenarios. The effectiveness of this classification method may be compromised on datasets with highly imbalanced category sample counts, and its robustness in situations involving small samples or long-tail distributions still necessitates further enhancement. Moreover, validation was primarily conducted using commonly employed public datasets. Practical applications may face more intricate challenges such as varying lighting conditions, noise interference, and sensor discrepancies. Future research could investigate the integration of multi-scale feature extraction fusion, data augmentation techniques, or transfer learning strategies to bolster the model’s robustness and generalization capabilities.

## 5 Conclusion

We propose a DS-Mamba model for HSI classification, which mainly consists of a DSpaM, a DSpeM, a feature fusion module and an ECA classification module. By integrating depth-separable convolution with Mamba blocks, the model effectively captures remote dependencies with linear complexity while simultaneously reducing both the number of parameters and computational requirements, and the incorporation of ECA attention significantly enhances the model’s performance. Experimental results across three commonly used datasets demonstrate the effectiveness of the proposed model, achieving high classification accuracy while also lowering computational complexity. Future work will strive to explore more applications of Mamba in hyperspectral image classification tasks and develop lightweight networks to further improve the classification accuracy.
